# Sore throat, fever, and pancytopenia during winter

**DOI:** 10.1002/ccr3.8800

**Published:** 2024-04-23

**Authors:** Jye Gard, Amy Howell, Claudia Boubeta, Hannah Corcoran, Fiona Bell, Kathryn McMahon

**Affiliations:** ^1^ Department of Paediatrics Werribee Mercy Hospital Werribee Victoria Australia; ^2^ The Department of Paediatrics, Melbourne Medical School Notre Dame University Werribee Victoria Australia; ^3^ The Royal Children's Hospital Melbourne Parkville Victoria Australia; ^4^ The Department of Paediatrics, Melbourne Medical School University of Melbourne Melbourne Victoria Australia

**Keywords:** acute medicine, adolescent medicine, infectious diseases, oncology, paediatrics

## Abstract

Care must be taken to mitigate the effect of cognitive bias in times of frequent common presentations. The etiology of bicytopenias and pancytopenias must always be carefully investigated. Blast cells in low count B ALL may not be seen on a peripheral smear and diagnosis often requires confirmational bone marrow aspirate with flow cytometry and molecular typing.

## 1 INTRODUCTION

We are all familiar with the surge of viral illnesses that hits emergency departments in winter. Thus, the presenting complaint of a child with a sore throat, fever, and lethargy may not initially spark much concern. This report, however, aims to demonstrate the importance of avoiding cognitive biases when met with seemingly “common” presentations during busy peak seasons. We present the case of a previously well 9‐year‐old girl who presented with a 2‐day history of syncopal episodes with associated fever and coryza. We also highlight some of the diagnostic challenges we encountered namely in the interpretation and selection of investigations.

## 2 CASE HISTORY/EXAMINATION

A previously healthy 9‐year‐old girl presented to an outer metropolitan emergency department (ED) with a 2‐day history of syncopal episodes, on a background of 8 days of fevers, lethargy, and pallor. During the preceding 4 weeks, she had also been ill with both recurrent tonsillitis and COVID‐19, associated with sore throat, cough, and coryza. She had a brief period of recovery, returning to school shortly before becoming unwell again and presenting to emergency. In ED, she was found to be febrile with all other observations within normal parameters and was assessed as moderately dehydrated requiring intravenous fluid support. On review by the pediatric team, examination showed significant pallor and slightly enlarged tonsils with no exudate. The rest of the examination was unremarkable with no overt lymphadenopathy, rashes or signs of bleeding.

## 3 INVESTIGATIONS

The patient was admitted to the pediatric ward for further workup including full blood examination (FBE), inflammatory markers, blood cultures, viral PCR, urine MCS, and ECG. A FBE was collected thrice via venepunctures as the laboratory rejected the first two samples due to suspected iatrogenic dilution of the collected blood. The third blood sample, however, continued to show a severe pancytopenia with a normochromic microcytic anemia, hemoglobin 43 g/dL, mean corpuscular volume 78 fL, mean corpuscular hemoglobin 28 pg, platelets 2 × 10^9^/L, WCC 1.3 × 10^9^/L, and neutrophil count 0.0 × 10^9^/L. Other blood results showed mildly deranged liver function tests (LFTs), hypoalbuminemia (25 g/L), and C‐reactive protein of 262 mg/L.

## 4 TREATMENT

Intravenous piperacillin/tazobactam was commenced to treat neutropenic sepsis, and a hemolysis screen sent. The patient was transfused with one unit of platelets and packed red blood cells. On the second day of hospitalization, the blood film returned showing rare tear drop cells and a single cell with high nuclear‐cytoplasmic ratio suspicious of a blast. Blood culture grew a gram‐negative bacillus, later confirmed as *Pseudomonas aeruginosa*. The patient was transferred to a tertiary pediatric hospital under hematology for further investigation including a bone marrow aspirate. These tests revealed average‐risk B‐cell acute lymphoblastic leukemia.

## 5 DISCUSSION

Syncope is defined as a brief, transient, and sudden loss of consciousness resulting from a temporary disruption to cerebral function.^1^ It is not an uncommon presenting symptom in the pediatric population, with one fifth of children expected to experience syncope by the end of adolescence. In most cases, the cause is benign, being autonomic or neurally mediated in nature.^1^ However, this case reinforces the importance of avoiding early cognitive bias and consideration a wide range of possible differentials for syncope particularly in premenarche children (Table 1).^2^ A thorough history and examination is crucial in discriminating between benign and serious etiologies. An ECG should always be performed and further initial tests for consideration include a blood glucose level, FBE, and pregnancy testing, depending on the clinical scenario.

**TABLE 1:** Syncope differential diagnoses.

Autonomic	Vasovagal, for example, cough, micturition, defecation, and heat related
	Orthostatic hypotension
	Postural orthostatic tachycardia syndrome
	Breath‐holding spells
Cardiac	Bradyarrhythmias (e.g., heart block)
	Tachyarrythmias (e.g., SVT)
	Long QT syndrome
	Brugada syndrome
	Wolff–Parkinson–White syndrome
	Infective (e.g., myocarditis and pericarditis)
	Structural abnormalities (e.g., congenital heart disease, ventricular outflow obstruction, aortic stenosis, cardiomyopathy, and pulmonary hypertension)
Other differentials	Anemia
	Hypoglycemia
	Functional disorder
	Seizure
	Vertigo
	Migraine
	Narcolepsy
	Toxic exposure (e.g., carbon monoxide and clonidine)
	Pregnancy

Of note in this case, the patient presented with syncope, pallor, and lethargy, suggestive of anemia, for which we considered an FBE as an important first line investigation. We are all too familiar, however, with difficulties obtaining a full blood count result in the pediatric setting. This is most often due to a pre‐analytical issue, such as a clotted or insufficient sample.^3^ Given how common this is, a result of “needs recollection” might not be given much additional thought.^3^, ^4^ However, it is important to consider alternative issues. Modern automated hemanalyzer machines may flag low cell counts as unreliable, prompting a repeat sample request only rather than release of critical results.^3^, ^4^ For example, diluted samples (e.g., samples taken from an intravenous line) may result in falsely low white cells, red blood cells, hematocrit, and platelets. The clinician will be required to decide whether to repeat the sample or not based on the clinical scenario. In this case, three repeat FBE samples were obtained due to our level of clinical concern. Therefore, another key learning point of this case is that if a pancytopenia is suspected, laboratory services should be notified, and blood film examined by a senior clinician.

As illustrated in Section 2, our clinical suspicions of anemia were confirmed on the final FBE result which showed a broader pancytopenia. Anemia and pancytopenia are again not uncommon entities among the pediatric population. Pancytopenia is the triad of anemia, leukopenia, and thrombocytopenia, and describes a decrease in all peripheral blood lineages below the normal reference range.^5^ Importantly to note, pancytopenia is not a diagnosis, but a manifestation of an underlying disease process with an extensive range of possible etiologies (Table 2).^5^, ^6^

**TABLE 2:** Causes of pancytopenia in children.^5^, ^6^

Decreased bone marrow function	Leukemia
	Bone marrow lymphoma
	Bone marrow metastases
	Myelodysplasia
	Aplastic anemia
	Fulminant sepsis
	Fanconi anemia
	Shwachman–Diamond syndrome
	Dyskeratosis congenita
	Megaloblastic anemia
	Viral infections (transient bone marrow suppression)
Increased destruction or sequestration of blood cells	Hypersplenism
	Sepsis
	HIV, hepatitis infections, EBV, and CMV
	Malaria, leishmaniasis, and filariasis
	SLE
	Paroxysmal nocturnal hemoglobinuria
	Hemophagocytic lymphohistiocytosis (HLH)

It is crucial therefore, as this case highlights, to have a systematic approach to investigating all pancytopenias. LFTs, viral serology, coagulation studies, direct antiglobulin test, B12, and folate may also be useful in further workup of pancytopenia.^7^, ^8^ Additionally, bone marrow aspiration and biopsy is indicated in most cases of pancytopenia.^8^ Diagnostic evaluation includes cytology (e.g., megaloblastic changes, dysplastic changes, and abnormal cell infiltrates), immunophenotyping (e.g., leukemias and lymphoproliferative disorders), and cytogenetics (e.g., myelodysplasia, leukemias, and lymphoproliferative disorders).

In children, one of the most common causes of pancytopenia is leukemia, most frequently acute lymphoblastic leukemia (ALL), where 8%–12% of ALL cases present with pancytopenia. In leukemia, bone marrow findings can be hypocellular or hypercellular.^9^ In hypocellular low‐count leukemia, blasts may go undetected on peripheral blood film due to low‐circulating populations. Hence, ALL should still be considered in pancytopenia even when peripheral blasts are not detected on blood film, as initially occurred in this case.^9^

Importantly, supportive care is the most important aspect of management in pancytopenia, and bleeding and infection in cytopenia should be considered a medical emergency and treated promptly according to local guidelines. To improve outcomes, it is recommended that empiric antibiotics be administered within 1 h of presentation with febrile neutropenia. This is reduced to 30 min if signs of shock are present. This is more challenging in cases where febrile neutropenia is the presenting feature of an oncological diagnosis.

Overall, this case is an important reminder to maintain a high index of suspicion for more sinister causes of symptomatic anemia with syncope even when confronted with a seemingly “common” presenting complaint.

## AUTHOR CONTRIBUTIONS

**Jye Gard:** Project administration; writing – original draft; writing – review and editing. **Amy Howell:** Writing – original draft. **Claudia Boubeta:** Writing – original draft. **Hannah Corcoran:** Writing – original draft. **Fiona Bell:** Writing – original draft. **Kathryn McMahon:** Writing – original draft.

## FUNDING INFORMATION

None.

## CONFLICT OF INTEREST STATEMENT

The authors have no conflict of interest to declare.

## ETHICS STATEMENT

Informed consent was obtained from the patient's parent to publish this report in accordance with the journal's patient consent policy.

## CONSENT

Written informed consent was obtained from the patient's parent to publish this report in accordance with the journal's patient consent policy.

## Data Availability

Data sharing is not applicable to this article as no new data were created in this case report.
